# A simple strategy for addition of degron tags to endogenous genes harboring prior insertions of fluorescent protein.

**DOI:** 10.17912/micropub.biology.000622

**Published:** 2022-08-09

**Authors:** Razan Fakieh, Tam Duong, You Wu, Neal Rasmussen, David Reiner

**Affiliations:** 1 Texas A&M University

## Abstract

There exist insufficient validated “entry portal” sites in the *C. elegans* genome for CRISPR/Cas9-dependent insertion into endogenous genes to confer diverse spatiotemporal patterns and levels of expression on exogenous sequences. Consequently, we recognized the most common potential “entry portal” sequences: genes previously tagged with fluorescent proteins using CRISPR/Cas9. As proof of concept, we used existing mKate2-encoding sequences inserted in the 5’ end of genes as an insertion point for the auxin inducible degron, AID*. This sequence permits reasonably efficient insertion that can be employed using a variety of approaches for different end goals. Our strategy is thus generalizable to many needs.

**Figure 1. Insertion of AID* into the 5’ end of mKate2 .**
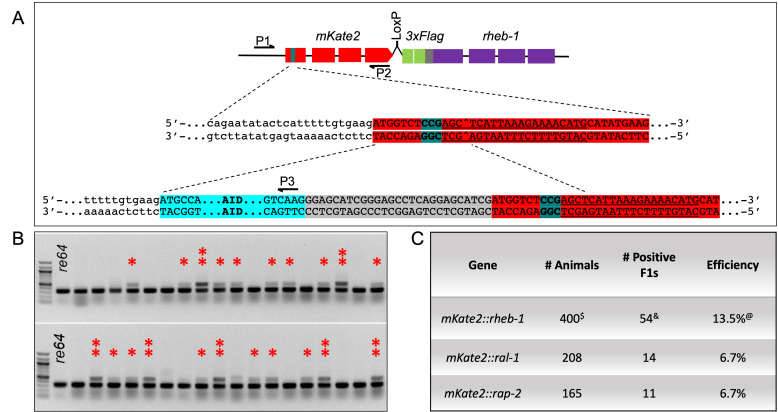
**A)** A schematic of AID* insertion into *mKate2::3xFlag::rheb-1* . Red depicts sequences encoding mKate2, cyan depicts sequences encoding AID*, teal indicates PAM sequences on the reverse strand, underlining indicates the guide RNA on the reverse strand with the carat symbol indicating the cut site, green depicts sequences encoding 3xFlag, gray depicts sequences encoding linker, and purple depicts the *rheb-1* gene. P1, P2 and P3 indicate locations of triplex PCR detection primers. **B)** The agarose gel for genotyping AID* insertion into *mKate* -tagged *rheb-1* . *re64* is the original *mKate2::3xFlag::rheb-1* strain, which yields a 337 bp band. AID* insertion bands are at 499 bp. Pools of four F1 animals were on each plate and in each PCR tube. One asterisk indicates a likely insertion event in one of the eight homologous chromosomes present in the pool of four co-CRISPRed F1 animals. The two vertical asterisks marking darker insertion bands indicate likely insertion events in two or more chromosomes, either as a homozygous insertion or heterozygous in two different animals. Pools of four were only used for insertion into *mKate2::3xFlag::rheb-1* . **C)** Table portraying efficiency of insertion. For each *mK2::gene* , the number of Rol animals picked, number of positive F1 candidates and efficiency rate for each gene are shown. ^$^ Total number of animals picked in pools of 4, equaling 100 reaction/400 F1 animals. ^&^ Estimate of positive F1 candidates from *mK2::rheb-1* pools. ^@^ Estimate of the efficiency rate for AID insertion into *mKate2::3xFlag::rheb-1* .

## Description

Genome editing with CRISPR in association with the bacterial nuclease Cas9 (“CRISPR/Cas9”) is used experimentally in many organisms and is under development for therapeutic applications. CRISPR has been applied successfully in *C. elegans* in a wide variety of contexts, from simple gene knockouts and knockins of missense and nonsense mutations to more elaborate tagging of genes, chromosomal rearrangements and constructs for a wide variety of applications, ( *e.g.* Ashley et al., 2021; Dejima et al., 2018; Dickinson et al., 2015; Dickinson et al., 2013; Friedland et al., 2013). Additionally, over the recent years various improvements in efficiency of CRISPR workflows have improved the potential for *a la carte* editing of the *C. elegans* genome (Arribere et al., 2014; Dokshin et al., 2018; Ghanta & Mello, 2020; Kim et al., 2014; Paix et al., 2015; Paix et al., 2016; Paix et al., 2014). Yet efficiency is still determined in part by selection of the 20 nt target sequences immediately upstream of the PAM (protospacer adjacent motif) for recognition by the Cas9 ribonucleoprotein complex. Selection of this target sequence during the design phase of an editing project determines synthesis of CRISPR RNAs (crRNAs or “guides”) for subsequent edits.

However, there persists great variability in efficiency of cutting, for example due to position in the gene or local chromatin context in the tissue in which cutting occurs (Chen et al., 2017; Ghanta & Mello, 2020; Horlbeck et al., 2016; Strohkendl et al., 2021; Yarrington et al., 2018), *i.e.* the distal gonad in *C. elegans* . These variables can reduce efficiency of editing projects or even prevent successful editing. One solution for this problem is to use validated “entry portals” for inserting large sequences. These sites could be either introduced exogenously into the *C. elegans* genome or using defined endogenous sequences previously demonstrated to be effective for insertions (Silva-Garcia et al., 2019; Stevenson et al., 2020). Alternatively, one could use “entry portal” sites serendipitously generated as part of CRISPR insertion projects for other ends, such as tagging endogenous genes with sequences encoding fluorescent proteins (FPs), epitopes for detection by antibodies, or other manipulations.

We identified the 5’ end of sequences encoding the mKate2 red fluorescent protein, used to tag endogenous genes to track proteins visually, as such a potential “entry portal” sequence to be used frequently for other applications (see Methods). Our lab has generated tags of three independent genes at the 5’ end with germline optimized (GLO; Fielmich et al., 2018) *mKate2* -encoding sequences. We have identified a guide RNA site in the 5’ end of the *mKate2* sequence that facilitates efficient insertion of desired sequences. We then used this site to insert a chemical genetic tool, the conditional auxin inducible degron (AID*; Zhang et al., 2015), into *mKate2* sequences already present in the genome. These sites mediate insertion at levels of efficiency likely to support generalized use of *mKate2* insertions as entry portals for editing into any gene previously modified by insertion of sequences encoding mKate2.

Specifically, we edited the 5’ ends of endogenous genes *ral-1* , *rheb-1* and *rap-2* , all of which encode small GTPases of the Ras family. All three had sequences encoding *mKate2^3xFlag* inserted at the 5’ end (Duong et al., 2020; Shin et al., 2018; Fakieh and Reiner unpublished results) using the SEC approach (Dickinson et al., 2015). We identified a possible guide sequence near the 5’ end of *mKate2* as a candidate insertion site ( **Fig. 1A** ). Using PCR oligonucleotides with homology arms matching the surrounding *mKate2* sequences, we PCR amplified AID* sequences from plasmid pBS::AID::mkate2::2xHA::AID * (see Methods). This amplicon was used to repair into the endogenous *mKate2* -inserted locus cut with Cas9 (see Methods). Successful editing into this targeted site resulted in AID* insertion into *mKate2^3xFlag::rheb-1* with substantial efficiency: conservatively, ~13.5% of *mKate2::rheb-1* animals had AID* insertions in the candidate site at the 5’ end of *mKate::3xFlag* sequence inserted in *rheb-1* ( **Fig. 1B** ). AID* was inserted into *mKate2^3xFlag::ral-1* and *mKate2^3xFlag::rap-2* with 6.7% efficiency rate for both ( **Fig. 1C** ).

The *rheb-1* and *ral-1* genes are essential for *C. elegans* development. The originally characterized mKate2-tagged insertions for these two genes were validated by Western blot and assessment of function (Duong et al., 2020; Shin et al., 2018). *rap-2* , in contrast, is not essential for viability (Pellis-van Berkel et al., 2005). Thus, the *mKate::3xFlag* tag into *rap-2* has not been validated functionally. For all three genes, the proper insertion of AID* was confirmed by sequencing regions that had undergone homology-directed repair (HDR).

From this study we can extrapolate that efficiency of insertion in this mKate2 site is high enough to be used for introduction of desired sequences into the 5’ end of sequences encoding *mKate2* in diverse loci in the genome. We demonstrated proof of concept with insertion of AID* , but the approach can be generalized to other edits in the genome.

Diverse future applications follow from this observation. First, development of similar sites in the of 3’ sequences in *mKate2* ­-encoding sequences would facility for similar editing but at genes with *mKate2* -encoding sequences inserted at their 3’ ends, to keep inserted sequences distal from the native gene. Identification and validation at similar sites of other FPs, like eGFP, mNeonGreen, mTurquoise, mScarlet, etc., both at 5’ and 3’ ends, would maximize the potential for insertion at diverse points in the genome. Furthermore, selection of previously tagged genes with defined but limited expression patterns would facilitate expression of inserted sequences in those same limited tissues, particularly if the user employs 2A-related ribosomal skip peptides for independent protein expression from the same locus (Ahier & Jarriault, 2014). Similarly, genes with previously defined differences in expression levels could be tagged and used as insertion points, which could confer similar control on independent exogenous sequences inserted at the locus. For example, while in some cases, like heat shock-dependent expression (Russnak & Candido, 1985), extremely high protein levels might be desirable, often physiologically relevant gene expression might be more desirable and demands can vary by experiment. These approaches would be limited only by the availability and characterization of previously tagged genes, but the size of this list is increasing exponentially.

In summary, we defined an efficient insertion site via CRISPR in the 5’ end of *mKate2* -encoding sequences inserted into endogenous genes. We demonstrated successful insertions of AID*-encoding sequences into the 5’ ends of three genes, *rheb-1* , *ral-1* and *rap-2* . This efficiency establishes feasibility for additional edits in different parts of the genome. With sufficiently high efficiency, researchers should be reasonably confident of inserting any sequence desired. Additionally, this method will facilitate the use of diverse endogenous promoters in the animal, imposing a variety of conditions for heterologous expression.

## Methods

Strains : Strains were grown with OP50 bacteria on NGM agar plates at 20°C unless otherwise stated. All strains used in this study are listed in ( **Table 1** ) and primers in ( **Table 2** ).

Selection of guide RNA sequence . The variability of the guide selection process was mitigated using certain general principles. First, we used a combination of algorithms at CRISPOR ( http://crispor.tefor.net/) and WU-CRISPR ( http://crisprdb.org/wu-crispr/ ) to evaluate potential guide RNAs and their uniqueness in the *C. elegans* genome (Doench et al., 2016; Wong et al., 2015). Second, in general we favor use of a “GG” dinucleotide at positions -1 and -2 upstream of the PAM (Farboud & Meyer, 2015), but that requirement was not met for the guide RNA used in this study and efficiency was still reasonable.

Preparation of repair template : AID* repair template was amplified by PCR from plasmid pBS::AID*::mKate2::2xHA::AID with germline optimized sequences (Neal Rasmussen, personal communication, DNA sequence available upon request). PCR primers had 5’ end homology arms matching sequences flanking the Cas9 cut site in *mKate2* to facilitate HDR ( **Table 2** ). The resulting PCR products were evaluated by gel electrophoresis and column purified using the Invitrogen PCR Purification Kit. Resulting purified repair template was added to the injection mix at the appropriate concentration (see below).

Preparing components for CRISPR : The composition of the injection mix was derived from a number of previous publications (Dokshin et al., 2018; Paix et al., 2015; Paix et al., 2016; Paix et al., 2014). The injection mix is presented in tabular form, below, and is assembled as two separate mixes, the Cas9/RNA complex and the DNA repair mix. All reported final concentrations are in reference to the final combined 20 μl injection mix.

**Table:** 

	**Injection Mix**	**Final Conc. - Injection Mix (20 μl)**	
**Cas9**	1 μl of 5 μg/μl	0.25 μg/μl	Incubate at 37°C for 15 min
**tracrRNA**	1 μl of 2 μg/μl	0.1 μg/μl	
**dpy-10 crRNA**	1.4 μl of 0.4 μg/μl	0.028 μg/μl	
**gene-specific crRNA**	1.4 μl of 0.4 μg/μl	0.028 μg/μl	

**Table:** 

**dpy-10 ssODN repair**	3.3 μl from 20 μM	3.3 μM = 2 μg = 100 ng/μl	
**gene-specific ssODN repair**	6.6 μl from 10 μM	3.3 μM = ? μg = ? ng/μl	
**or**			
**gene-specific PCR product repair**	2 μg	100 ng/μl	Incubate according to Mello Donor PCR stepdown below
**ddH20**	up to 20 μl	----------	

**Do not add PCR repair template to RNP mix until Mello donor program has been run**

To identify animals in which CRISPR editing had occurred in a gamete from the parents, we used the *dpy-10(cn64* gf *)* semi-dominant co-CRISPR marker phenotype, with repair template co-injected as a single-stranded DNA oligonucleotide (Arribere et al., 2014). Independently of the enzyme mix, repair template for AID* insertion was made using two different protocols. First, for insertion into *mKate2::3xFlag::rheb-1* , two PCR templates of AID* were amplified, with and without homology arms matching flanking *mKate2* sequences. The two PCR products were mixed at 0.4 μg/ml and subjected to a melting-annealing program to facilitate formation of “bubbles” due to incomplete reannealing (Ghanta et al., 2021; Ghanta & Mello, 2020). The program was as described (Ghanta et al., 2021): 95˚C 2:00 min; 85˚C 10 sec.; 75˚C 10 sec, 65˚C 10 sec, 55˚C 1:00 min, 45˚C 30 sec, 35˚C 10 sec, 25˚C 10 sec, with ramp down increments of 1˚C/sec, then held at 4˚C.

Second, for insertion into *mKate2::3xFlag::ral-1* and *mKate2::3xFlag::rap-2* , we amplified AID* with homology arms, without the added step of generating a heteroduplex from two different PCR products. For this second approach, 3.3 μM final concentration of the AID* PCR product was subjected to the same melting-annealing program described above.

For both approaches, 3.3 μM final concentration of *dpy-* *10* single-stranded oligonucleotide repair template was added to the PCR product, the final 15.2 μl in nuclease free water, added to the 4.8 μl of ribonucleoprotein complex for a final volume of 20 μl and very gently mixed without vortexing. This mix was filtered through a 0.45 μm cutoff ultrafiltration spin-columns (EMD Millipore Corp.) for 2 minutes at 13,000 rpm.

Injection and isolation : We injected mid-adult animals with no greater than one row of embryos in the uterus. P _inj_ animals were picked singly to spotted plates and incubated at 20 °C for ~96 hrs. The oldest F1 animals, corresponding roughly to the first 24 hrs of egg laying, were passed over. From the younger cohort of F1s, we picked animals expressing the Rol co-CRISPR marker singly to plates, with the exception of *rheb-1* , which were picked in pools of four. The next day, parents were picked into tubes and lysed for single-worm PCR with triplex detection primers (Table 2). PCR products were run on 2% agarose gels. Homozygotes segregated from positive F1 parents were isolated from singly cloned F2 non-Rol animals, then tested and confirmed by PCR.

## Reagents

Table 1: List of Strains used in this study

**Table:** 

	
Strain	Genotype
DV3066	*rheb-1(re64[mKate2^3xFlag::rheb-1]) III*
DV3668	rheb-1(re64 re283[AID*::mKate2^3xFlag::rheb-1]) III
DV3402	*ral-1(re218[mKate2^3xFlag::ral-1]) III*
DV3814	ral-1(re218 re319[AID*::mKate2^3xFlag::ral-1]) III
DV4012	*rap-2(re346[mKate2^3xFlag::rap-2]) V*
DV4021	rap-2(re346 re355[AID*::mKate2^3xFlag::rap-2]) V

Table 2: List of DNA and RNA oligonucleotides used in this study

**Table:** 

Name	Description	Sequence ^a^
tracrRNA	tracer RNA	AACAGCAUAGCAAGUUAAAAUAAGGCUAGUCCGUUAUCAACUUGAAAAAGUGGCACCGAGUCGGUGCUUUUUUU
TD332	*mKate2* crRNA	CAUGUUUUCUUUAAUGAGCUCGG + GUUUUAGAGCUAUGCUGUUUUG
*dpy-10*	*dpy-10* crRNA	GCUACCAUAGGCACCACGAG + GUUUUAGAGCUAUGCUGUUUUG
*dpy-10 rep*	Repair template for *dpy-10* co-CRISPR	CACTTGAACTTCAATACGGCAAGATGAGAATGACTGGAAACCGTACCGCATGCGGTGCCTATGGTAGCGGAGCTTCACATGGCTTCAGACCAACAGCCTAT
TD326	AID* (for) repair template in *rheb-1* with homology arm	tcaatcattcaatattcagaatatactcatttttgtgaag ATGCCAAAGGACCCAGCTAA
TD327	AID* (rev) universal repair template with homology arm in *mKate2* ^b^	CCCTCCATGTAGAGCTTCATATGCATGTTTTCTTTAATGAGCTCGGAG ACCATCGATGCTCCTGAGG
TD328	AID* (for) repair template without homology arm	ATGCCAAAGGACCCAGCTAA
TD329	AID* (rev) repair template without homology arm	ACCATCGATGCTCCTGAGG
TD330	genotyping (for) *rheb-1*	gcgcatattattgcaaaagtacatc
TD331	genotyping (rev) (in *mKate* 2) ^c^	CCTCGACGGCCTTGATAC
YW10	AID* (for) repair template in *ral-1* with homology arm	tttcccccgggttttccccctaaaactcgaattttcagcc ATGCCAAAGGACCCAGCTAAG
YW11	genotyping (for) *ral-1* in promoter	tcagaatggagggttacggt
YW59	genotyping (for) *ral-1* in AID*	GCCACCAGTTCGTTCTTACC
RF89	AID* (for) repair template with *rap-2* homology arm	cacctaatccaatttattgattttcagcaaaaaactctca ATGCCAAAGGACCCAGCTAA
RF91	genotyping (for) *rap-2* in promoter	gctactccactttagaatcaatttcagc
RF92	genotyping (rev) *rap-2* in AID*	CGGTAAGAACGAACTGGTGGC
RF93	genotyping (rev) *rap-2* in *mKate2*	GCGAATGGGAGTGGTCCTC

^a^ For primers for PCR of repair templates, seed primers are underlined and the chimeric sequences of homology arms not underlined

^b^ This reverse primer is universal for all insertions at this locus because the seed primer recognizes AID* and the homology arm is in *mKate2* . Because repairs neighbor promoter sequences at the 5’ end, a different homology arm will be needed for each 5’ forward primer for amplification of *AID* *.

^c^ For AID* insertion into *mKate2::3xFlag::rheb-1* only two detection primers were used, flanking AID* in the *rheb-1* promoter and in *mKate2* . But TD331(rev) was also used in triplex PCR to detect insertions into *mKate2::3xFlag::ral-1* and could theoretically be used for other insertions into *mKate2* -encoding sequences.
